# Integrated microbiome and metabolome analysis reveals mechanisms of *Fusarium* wilt resistance in banana rhizosphere soil under different continuous cropping durations

**DOI:** 10.3389/fmicb.2026.1853743

**Published:** 2026-10-22

**Authors:** Ming Zhao, Tianli Mo, Xiang Huang, Haiwang He, Fang Long, Peng Wu, Yu Zou

**Affiliations:** Institute of Biotechnology, Guangxi Academy of Agricultural Sciences, Nanning, China

**Keywords:** banana, biocontrol microorganism, *Fusarium* wilt, metabolome, microbiome, root secretions

## Abstract

**Introduction:**

*Fusarium* fungi are the primary causal agents of banana *Fusarium* wilt disease. In this study, using a resistant banana line (derived from cultivar Baodao), we observed that with increasing years of continuous cropping, the incidence of *Fusarium* wilt disease showed a notable reduction.

**Results:**

Microbial community abundance was lower in the susceptible banana variety (V) than in the resistant variety [sampled after one (S1), two (S2), and three (S3) years of continuous cropping]. With increasing continuous cropping years (S1, S2, and S3), microbial abundance increased, while the abundance of *Fusarium oxysporum* significantly decreased, and these changes coincided with visually healthier plant growth compared to the susceptible variety. However, the relative abundance of *Fusarium*, *Bisifusarium Gibellulopsis*, and *Rectifusarium* were the highest in V. Rozellomycota sp., *Trichoderma virens*, *Trichoderma asperellum*, and Hypocreaceae sp. were also identified with high abundance in S2 and S3, but were not detected in V. In addition, using Spearman’s correlation analysis and network analysis (|r| > 0.6), we found that the reduction of *Fusarium* pathogens was significantly correlated with the enrichment of beneficial bacteria such as *Sphingomonas parvus* and Rozellomycota sp. A total of 1153 metabolites were identified, including amino acids, phenolic acids, organic acids, phenolic acids, and flavonols. Among these, 79 compounds showed a significant decrease in V but increased in S1, S2, and S3; these compounds were negatively correlated with *Fusarium oxysporum* and positively correlated with Rozellomycota sp. Additionally, 47 compounds were associated with *Sphingomonas parvus*, *Bryobacter* and other beneficial bacteria.

**Conclusion:**

These findings suggest that specific metabolic compounds are associated with the enrichment of beneficial fungi and bacteria in the banana rhizosphere soil as continuous cropping time increases.

## 1 Introduction

The edible dessert bananas, the most-selling fresh fruits in the world, are classified as part of “Magnoliophyta,” class “Liliopsida,” order “Zingiberales,” family “Musaceae,” and genus “*Musa*,” which is also one of the earliest cultivated main economic food crops (Qamar and Shaikh, 2018; Zou et al., 2022). Bananas can be easily added to a regular diet by simply eating fresh fruits or by adding them to other foods, such as yogurt or smoothies, due to their unique sweet taste and flavor. In African regions, plantains comprise a significant and essential component, contributing considerably to food security and income generation for more than 70 million Africans (Viljoen et al., 2020). Therefore, bananas are a globally frequently consumed fresh fruit. Bananas are mainly cultivated in China, India, Indonesia, America (Hawaii), Brazil, Africa, Ecuador, and Philippines. Statistics show that in the past two decades, banana and plantain production has continued to expand, reaching about 160 million tons in 2021 (Rocha et al., 2021; Zou et al., 2022). The banana and its subgroups are the most traded and exported fruit globally, accounting for about 20% of global production (FAO, 2020), which is an important economic pillar for many tropical and subtropical countries (Rocha et al., 2021). The banana crops are of great importance to local food and regional food security (Dita et al., 2011).

Bananas are high in critical nutrients such as vitamins, potassium, carotenoids, manganese, fiber, and dopamine, which can help with hypertension, cancer, diabetes, and depression (Pereira and Maraschin, 2015; Singh et al., 2016). The health benefits associated with the consumption of bananas and related processed products are highly correlated with their essential nutritional contents (Sulaiman et al., 2011), whereas banana quality, yield, composition, and growth development are influenced by their origin and related growing conditions. Climate (prolonged wet or dry circumstances, temperature extremes, storm damage), soil (poor soil drainage and aeration, unfavorable chemical or physical conditions), and infections all have a significant impact on the disease (Thangavelu et al., 2022). Among them, soil-borne pathogens (*Fusarium oxysporum*, *Xanthomonas vasicola*, *Pseudocercospora fijiensis*, etc.) are important factors causing wilt, yield reduction, and quality decline in bananas and other plants. Continuous cropping could cause significant changes in rhizosphere microbial community of banana soil. Dysbiosis, or the unbalance in microbial communities caused by the growth of pathogenic bacteria, is frequently linked to illness (Molina et al., 2022). Plant roots and soil microbial communities interact complexly, according to earlier research (Letuma et al., 2018). Plant root-associated microbial communities, including endosphere, rhizoplane, rhizosphere and soil, aid plants by preventing pathogenic infection and assisting in the uptake of nourishment from the soil (Chen et al., 2022). Therefore, considering the popularity, health benefits, and economic importance of bananas to humans, it is necessary to explore the interaction between the plant growth and continuous cropping of bananas and microorganisms such as pathogens.

Banana *Fusarium* wilt is one of the main fungal diseases in banana production, which is caused by the most serious soilborne fungus illness, *Fusarium oxysporum* f.sp. cubense Tropical race 4 (TR4) (Li et al., 2022). The pathogen is classified as a hemi-biotroph since it initially creates a biotrophic interaction with the host before transitioning to necrotrophy, in which host tissue is killed (Dita et al., 2018). In the decades since, TR4 has spread rapidly with a wide range of occurrences and has proven difficult to control, first in Southeast Asia but more recently in Africa, Australia, and Western Asia banana planting areas such as Hainan, Yunnan, Guangdong, Guangxi, and Taiwan in China, and in Columbia, Peru, etc., (Martínez et al., 2023; Ploetz, 2015). TR4 has strictly asexual reproduction, producing microconidia, macroconidia, and chlamydospores as survival structures, and has been discovered to invade the xylem tissues of roots and spread through the vascular system of pseudo stems, significantly reducing banana yield or even causing plant death (Pegg et al., 2019). In 1976, 1983, and 1999, respectively, half a million banana plants in China Taiwan were infected and dispersed over 1,200 ha, 1,500 ha, and 3,000 ha (Hwang and Ko, 2004).

Tropical race 4-infected banana plants exhibit yellowing young leaves, splitting false stems, and eventually mortality, severely reducing banana quality and yield (Lopes et al., 2014; Pegg et al., 2019). The pathogen can induce disease at low inoculum levels, can live at depth in the soil, and has a long incubation period (Pegg et al., 2019). Infections cause a host defense response that includes gels, tylosis, and lignification, which results in vascular blockage. Even susceptible cultivars exhibit this reaction (Carvalhais et al., 2019). Furthermore, the pathogen persists in soil or colonized host tissue by producing chlamydospores with a long survival potential, growing as hyphae in organic residues, and invading and surviving as asymptomatic endophytes in a variety of non-host plants (Hennessy et al., 2005; Pittaway et al., 1999). TR4’s disease cycle and host pathogen interactions, on the other hand, are little understood. Much more study is needed because there are still many challenges connected with developing and implementing containment measures (Buddenhagen, 2009). Therefore, more effective detection methods and insights into how TR4 disrupts microbial communities in host plants and their surrounding soil to disrupt the production and spread of the disease pathogen’s initial propagules are urgent priorities for TR4 control (Ordonez et al., 2015).

Although chemical fungicides are successful at controlling banana wilt and increasing output, they are hazardous to human health and pollute the environment. However, biological control reveals that microorganisms or their secondary metabolites can successfully control this disease and limit pathogen growth (Gang et al., 2013; Li et al., 2022). The biological control’s low efficacy is attributed to inherent factors in the dynamics of the disease’s primary inoculum, particularly the production of chlamydospores, which persist in cultivated areas, such as the ability to survive in crop remains as an endophytic fungus in alternative hosts (Warman and Aitken, 2018). Furthermore, the pathogen’s genetic diversity, which results in novel strains capable of infecting resistant cultivars, is a factor that limits the application of disease management and control approaches (Costa et al., 2015; Fourie et al., 2011). Therefore, in addition to conventional resistant cultivar breeding and the application of chemical fungicide treatments, using biocontrol bacteria, genetic engineering (genetic transformation) breeding, or mutation induction are also effective ways to make bananas resistant to TR4 (Wang et al., 2018).

After long-term banana planting and observation, we discovered that as the number of planting years increased, that correlated with a decrease in the incidence of TR4 disease in bananas. For example, 3-years-old banana plants are more resistant to TR4 disease than 1-year-old and 2-years-old plants. Therefore, we speculate that with the continuous cultivation of banana plants, there is a mutual regulatory effect between the microbial community in the soil and the banana root secretions, which increases the TR4 resistance in bananas. Therefore, based on this hypothesis, we selected the rhizosphere soil and root secretions of two banana groups: the TR4-susceptible variety (V) and the TR4-resistant variety at different continuous cropping stages–1 year (S1), 2 years (S2), and 3 years (S3)–which exhibited varying levels of resistance to *Fusarium* wilt. These samples were subjected to microbial diversity analysis (16S rRNA and ITS sequencing) and widely targeted metabolome detection, respectively. This study aims to explore how the rhizosphere microbial community and root secretion profiles of banana plants respond to TR4 across different continuous cropping years. The goal was to identify key secretory compounds and core microbiota (potential biocontrol agents) that may regulate and inhibit TR4 infection and reproduction, thereby contributing to the control of banana *Fusarium* wilt disease.

## 2 Materials and methods

### 2.1 Plant material and treatment

All plant and soil samples used in the present study were collected from Dengliu Village, Xianhu Town, Wuming District, Nanning City, and Guangxi Zhuang Autonomous Region (23.35° N, 108.09° E, altitude 700 m). This study employed the rhizosphere soil and root secretions of banana plants with different susceptibilities to *Fusarium* wilt and different continuous cropping histories. The susceptible variety (V) was Guijiao 6, in which the *Fusarium* wilt incidence rate exceeded 80% in the previous crop. The cultivar Baodao (tropical crop variety certification number: 2021010) is originally susceptible to *Fusarium oxysporum* f. sp. cubense Tropical race 4 (TR4). However, during continuous cropping over multiple years, a single resistant line (designated S) was selected from a Baodao population based on consistently low disease incidence (≤10% over two consecutive years) under natural infection conditions. The tissue culture banana seedlings (Baodao) were directly planted without rotation, with a row spacing of 1.8 m × 2.2 m, and the cultivation management was medium-level. This S line exhibited progressively enhanced resistance with increasing continuous cropping duration. From this S line, we sampled plants after one (S1), two (S2), and three (S3) years of continuous cropping. The genetic basis of this enhanced resistance is not yet fully understood and may involve epigenetic regulation or selection of naturally occurring resistant somaclonal variants, which is under further investigation.

To monitor *Fusarium* wilt occurrence, infected banana plants from the previous year were retained, and disease incidence data were collected over 3 years. *Fusarium* wilt was diagnosed based on typical symptoms, including yellowing of lower leaves, splitting of pseudostems, and vascular discoloration observed in cross-sections of the rhizome. Disease severity was assessed using a 0–4 grading scale: 0 = no symptoms; 1 = slight leaf yellowing affecting <10% of leaf area; 2 = moderate yellowing affecting 10%–25% of leaf area, with slight vascular discoloration; 3 = severe yellowing affecting 25%–50% of leaf area, with distinct pseudostem splitting and extensive vascular browning; 4 = plant wilting or death, with >50% leaf area necrotic. When infected plants were observed in the field, the number was recorded weekly until no new infections were detected for three consecutive weeks. Disease incidence was calculated as the number of diseased plants (severity grade ≥ 1) divided by the total number of planted plants per plot. The values presented in Table 1 represent the mean ± SD of three replicate plots per group. The three resistant banana groups–S1 (1 year), S2 (2 years), and S3 (3 years)–represent different continuous cropping durations from the same resistant variety. For each of the three replicate plots per group, five banana plants were randomly selected. From these, rhizosphere soil samples were collected as composite samples (five plants pooled per plot). Separately, root exudates and root tissues for DNA extraction were collected from a single representative plant per plot (the one showing the median disease severity score within the plot), yielding three independent biological replicates per treatment group. A total of 12 banana samples (4 groups × 3 replicates) were rapidly frozen in liquid nitrogen for 30 min and stored at −80 °C for subsequent DNA extraction, LC-MS/MS, and high-throughput sequencing.

**TABLE 1:** Field investigation of the phenotype of *Fusarium* wilt disease resistance in bananas at different cropping times.

Planting/cropping time	Disease incidence rate (%)
Susceptible (V) – 1 year	82.3 ± 5.6^a^
Susceptible (V) – 2 years	81.5 ± 4.2^a^
Susceptible (V) – 3 years	81.7 ± 6.4^a^
Resistant S1 – 1 year	24.6 ± 3.7^b^
Resistant S2 – 2 years	14.8 ± 2.3^c^
Resistant S3 – 3 years	5.3 ± 1.6^d^

*^a^*Different letters indicated the significant difference between the comparison groups (*p* < 0.05). The susceptible variety V was grown for 1 year only and is shown for reference.

### 2.2 Rhizosphere soil collection

In this study, rhizosphere soil from banana plants (shaking the root) grown in various years was collected by five-point sampling. Rhizosphere soil was collected by shaking roots vigorously to remove loosely adhering soil. The soil of each group was the rhizosphere soil of banana plants, that is, the rhizosphere soil samples were obtained by shaken off from the roots to remove impurities and through a 50-mesh sieve. Using sterile tweezers, the plant residue in the soil was extracted and placed into two sections of sterilized bags. Mixed samples are treated in the same way as soil samples from the rhizosphere, which were kept at −80 °C for DNA extraction and microbiome sequencing.

### 2.3 The rhizosphere microorganism detection in banana plants

Following the manufacturer’s instructions, the Fast DNA Spin Kit for Soil (MP Biomedicals, USA) was used to extract the whole microbial DNA from 1 g of soil samples. Total microbial DNA was extracted from each of the 12 composite soil samples (4 groups × 3 replicates) using the Fast DNA Spin Kit for Soil (MP Biomedicals, USA) according to the manufacturer’s instructions. Using 1% agarose gel electrophoresis and a Nano-Drop 2000 UV-vis spectrophotometer (Thermo Scientific, Wilmington, DE, USA), the concentration, purity, and purification of the DNA were observed. After that, the DNA was diluted to 1 ng/μL and stored at −80 °C in order to amplify the fungal internal transcribed spacer (ITS) gene. PCR amplification of the fungal ITS2 region was performed using primers ITS3_KYO2 (GATGAAGAACGYAGYRAA) and ITS4 (TCCTCCGCTTATTGATATGC). Bacterial 16S rDNA V3–V4 regions were amplified using primers 341F (CCTACGGGNGGCWGCAG) and 806R (GGACTACHVGGGTATCTAAT). Each 25 μL PCR reaction contained 12.5 μL of 2× Phusion High-Fidelity PCR Master Mix (New England Biolabs), 0.5 μM of each primer, and 20 ng of template DNA. Thermal cycling conditions were: initial denaturation at 98 °C for 30 s; 30 cycles of denaturation at 98 °C for 10 s, annealing at 55 °C (ITS) or 58 °C (16S) for 30 s, and extension at 72 °C for 30 s; followed by a final extension at 72 °C for 5 min. PCR amplicons were purified using the Trelief DNA Gel Extraction Kit (TSINGKE, TSP601-200, China) and 2% agarose gels according to the manufacturer’s instructions. Using an Illumina platform for paired-end sequencing (2 × 250) and pooling the purified amplicons in equimolar concentrations in accordance with established methodologies (Song et al., 2023).

### 2.4 ASV analysis of microbial community sequencing assembly

The amplicon sequence data were processed using the QIIME 2 platform (Bolyen et al., 2019). Briefly, the DADA2 plugin was employed for quality filtering, denoising, and merging of paired-end reads, and for the removal of chimeric sequences. This pipeline infers exact amplicon sequence variants (ASVs) with single-nucleotide resolution, providing a more accurate and reproducible measure of microbial diversity than traditional OTU clustering. Representative ASV sequences were taxonomically classified using a pre-trained Naive Bayes classifier against the SILVA database (for 16S) and the UNITE database (for ITS).

### 2.5 Metabolome detection of banana root exudate

Roots were washed five times with sterile deionized water. For each biological replicate, 5 g (fresh weight) of roots were placed in 50 mL sterile deionized water in a conical flask. Flasks were wrapped with aluminum foil to maintain darkness and incubated at 22 °C ± 2 °C for exactly 24 h. The exudate was then filtered sequentially through quantitative filter paper and a 0.45 μm membrane filter to remove debris and microorganisms. Filtrates were immediately frozen at −80 °C and vacuum freeze-dried. Freeze-dried samples were extracted with 70% methanol containing an internal standard (30× volume ratio), vortexed for 15 min, and ultrasonicated in an ice-water bath for 10 min. After centrifugation at 12,000 rpm for 3 min at 4 °C, the supernatant was filtered through a 0.22 μm membrane and stored in sample vials for LC-MS/MS detection.

Tandem mass spectrometry (Applied Biosystems 6500 Q TRAP) system (UPLC-MS/MS) and Ultra-Performance Liquid Chromatography (Shim-pack UFLC SHIMADZU CBM30A system) were used to evaluate the extract samples. The Liu et al. (2023) analytical conditions were adhered to for the HPLC column (Agilent SB-C18 1.8 μm, 2.1 mm × 100 mm), which measures 1.8 μm by 2.1 mm by 100 mm (Liu et al., 2023). The solvent system consists of ultrapure water and acetonitrile, both of which add 0.04% acetic acid. The gradient program includes five stages: 95:5 V/V (H_2_O/acetonitrile) at 0.00 min, 5:95 V/V at 11.0 min, 5:95 V/V at 12.0 min, 95:5 V/V at 12.1 min, and 95:5 V/V at 15.0 min. The flow rate is 0.40 mL/min, the temperature is 40 °C, and the injection volume is 2 μl. An alternative connection between the effluent and the ESI-triple quadrupole-linear ion trap (Q TRAP)-MS was made.

### 2.6 ESI-Q TRAP-MS/MS

A triple quadrupole-linear ion trap mass spectrometer (Q TRAP), an API 6500 Q TRAP LC-MS/MS system with an ESI Turbo Ion-Spray interface, was used to produce LIT and triple quadrupole (QQQ) scans. It is managed by the Analyst 1.6 program and is capable of operating in both positive and negative ion modes (AB SCIEX). The ESI source operating parameters were as follows: high collision gas (CAD), ion source gas I (GSI), gas II (GSII), and curtain gas (CUR) set at 55, 60, and 25.0 psi, respectively; ion source, turbo spray; source temperature 500 °C; ion spray voltage (IS) 5500 V (positive ion mode)/−4500 V (negative ion mode). Using 10 and 100 μmol/L polypropylene glycol solutions in QQQ and LIT modes, respectively, the device was adjusted and calibrated. Multiple reaction monitoring (MRM) trials with collision gas (nitrogen) set to 5 psi yielded QQQ scans. The DP and CE of each MRM ion pair were finished through further tuning of DP and CE. Based on the metabolites eluted during each interval, a specific set of MRM ion pairs was examined.

### 2.7 Statistical analysis of the data

Analyst 1.6.1 software and SPSS 20.0 were used to examine all metabolite data from banana samples. The means ± SD were calculated from these data. One-way ANOVA was used, followed by Tukey’s significant difference test, and *p*-values < 0.05 were considered significant. To identify differentially accumulated metabolites, orthogonal partial least squares-discriminant analysis (OPLS-DA) was performed. The reliability of the OPLS-DA model was evaluated using a 200-permutation test to prevent overfitting. The model was considered valid when the intercepts of R^2^ and Q^2^ regression lines were less than 0.4 and 0.05, respectively. The variable importance in projection (VIP) value was used to assess the contribution of each metabolite to the model. Metabolites with | log_2_ (fold change)| ≥ 1 and VIP ≥ 1 were considered differentially accumulated. R software^1^ was used to perform hierarchical clustering analysis of the metabolites between samples.

## 3 Results

### 3.1 The phenotype investigation of *Fusarium* wilt disease resistance

After many years of phenotypic identification of *Fusarium* wilt in banana plants, this experiment found that the resistance level (disease incidence rate) of *Fusarium* resistance banana varieties, such as Baodao banana, showed a downward trend with the increase of planting years (continuous cropping time) (Supplementary Figure 1). After plant 2 years, the susceptible banana cultivar easy by *Fusarium* infection. Cross-section of stem, longitudinal section of corm, and longitudinal section of stem show extensive vascular browning, necrosis, and tissue disintegration (Figure 1A). However, the resistant banana cultivar under the same conditions. All sections display intact vascular tissues, minimal discoloration, and healthy parenchyma, indicating effective resistance to *Fusarium* wilt (Figure 1B). By the second and third planting years, the *Fusarium* wilt disease incidence was reduced by about 40% and 78%, respectively, compared with first-year banana seedlings (Table 1). We then conducted follow-up experiments to detect and analyze the inhibition mechanism of rhizosphere soil microorganisms and root exudates against *Fusarium*.

**FIGURE 1:**
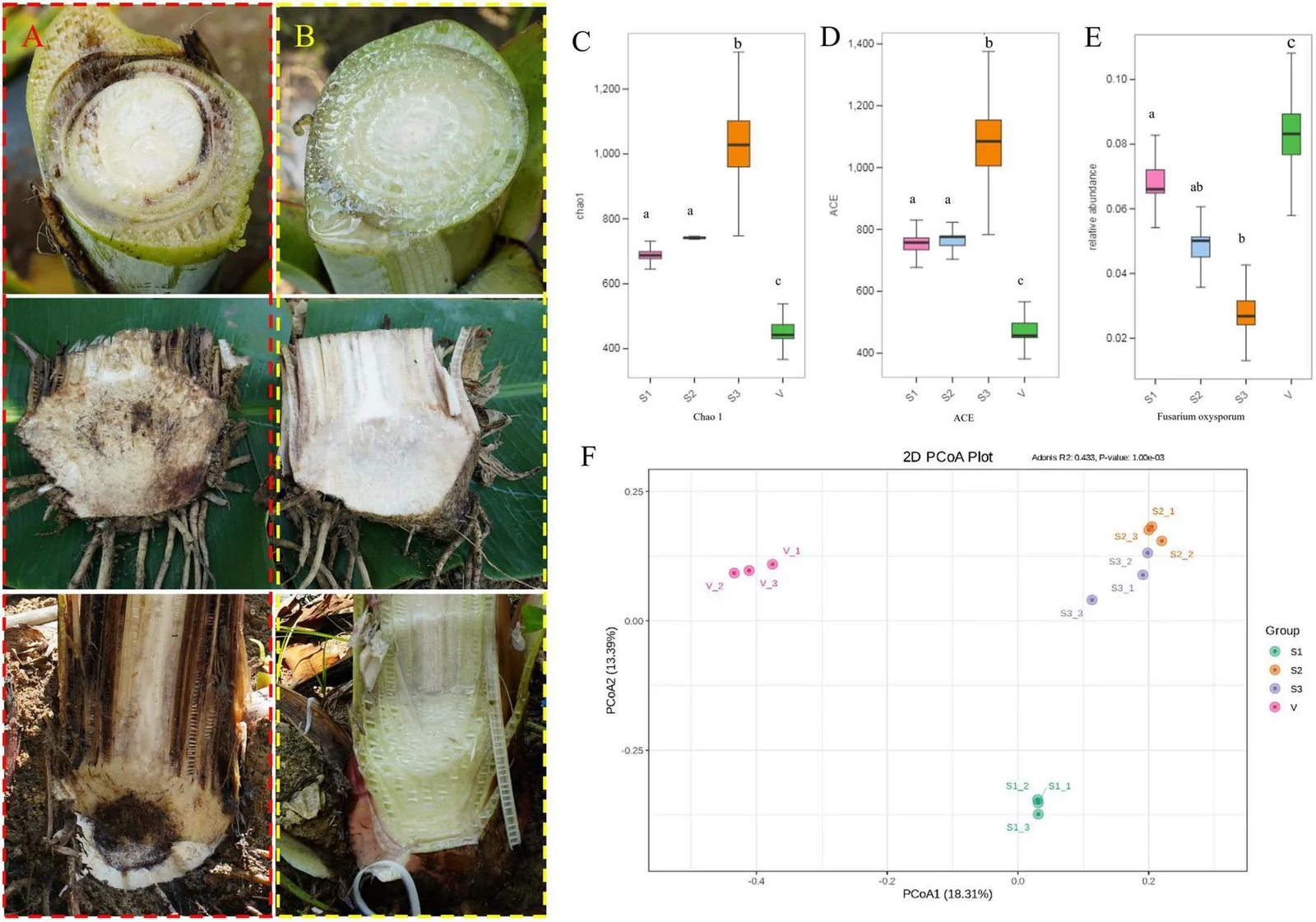
Phenotypes of banana plants 2 years post-inoculation with *Fusarium oxysporum*. **(A)** Susceptible cultivar (Guijiao 6); **(B)** Resistant cultivar (Baodao). For each panel, from left to right: stem cross-section, corm longitudinal section, and stem longitudinal section. Beta-diversity analysis of the microbial community diversity in each banana rhizosphere soil sample. Chao1 richness index **(C)** and ACE richness index **(D)** of *Fusarium* wilt fungal pathogens to impact on soil microbial diversity. Relative abundance of *Fusarium oxysporum* in each group **(E)**. Spearman’s correlation between *F. oxysporum* abundance and continuous cropping years: rho = –0.89, *p* < 0.001. **(F)** Principal coordinate analysis (PCoA) based on Bray-Curtis distances of fungal community composition. Different lowercase letters (a–c) indicate significant differences among groups (one-way ANOVA with Tukey’s HSD test, *p* < 0.05). V, susceptible variety Guijiao 6; S1, S2, S3, resistant line (derived from Baodao) after 1, 2, and 3 years of continuous cropping, respectively.

### 3.2 Analysis of microbial communities in the rhizosphere soil of different banana plants

Fungal and bacterial diversity in the rhizosphere soil of banana plants was detected and analyzed in the present study. A total of 1,532,708 effective tags were obtained in 12 banana root soil samples. Among them, each group obtained 906, 973, 977, and 1340 ASVs in V, S1, S2, and S3, respectively. At the same time, we used alpha-diversity to analyze the microbial community diversity (within-community) in each rhizosphere soil sample. Chao1 and ACE values showed that *Fusarium* wilt pathogens had a negative impact on soil microbial diversity (Figures 1C, D). The results of this experiment showed that the microbial diversity in V was the lowest. However, with the increase in planting time (year), the microbial richness in the soil also increased, and the banana planting soil environment at this time may gradually become healthy for plant growth. Before analyzing the overall fungal diversity, we first assessed the abundance of *Fusarium oxysporum* in each sample (Figure 1E). The results showed that the relative abundance of *Fusarium oxysporum* was the highest in the susceptible banana (V), reaching more than 0.8. In addition, the abundance of *Fusarium oxysporum* in S1, S2, and S3 was lower than that of V, and its abundance also decreased with the increase in continuous cropping (planting year). This is consistent with previous phenotypic observations (Table 1). Then, we conducted beta-diversity analysis for each soil sample. The results of PCoA analysis showed that V was clearly separated from other soil sample groups and had the farthest distance on PC1 (Figure 1F). S2 and S3 are relatively close to each other on PC1 (Figure 1F).

### 3.3 Changes in fungal abundance in banana rhizosphere soil at different planting ages

Combined with the results of the fungal abundance and significance analysis, a total of nine fungi with significantly different abundances were identified in different banana soil samples. The results show that in V, *Fusarium oxysporum*, *Bisifusarium delphinoides*, *Fusarium* sp., *Gibellulopsis piscis*, and *Rectifusarium ventricosum* have a very high abundance (Figure 2 and Supplementary Table 1). The abundance of these fungi decreased significantly with the banana continuous cropping years, reaching the lowest in S3. Therefore, the result suggested that these fungi may have a synergistic role in the pathogenesis of banana wilt. In addition, we also screened out 4 fungi strains with high abundance in S2 and S3, while their abundance was very low or even not detected in V.

**FIGURE 2:**
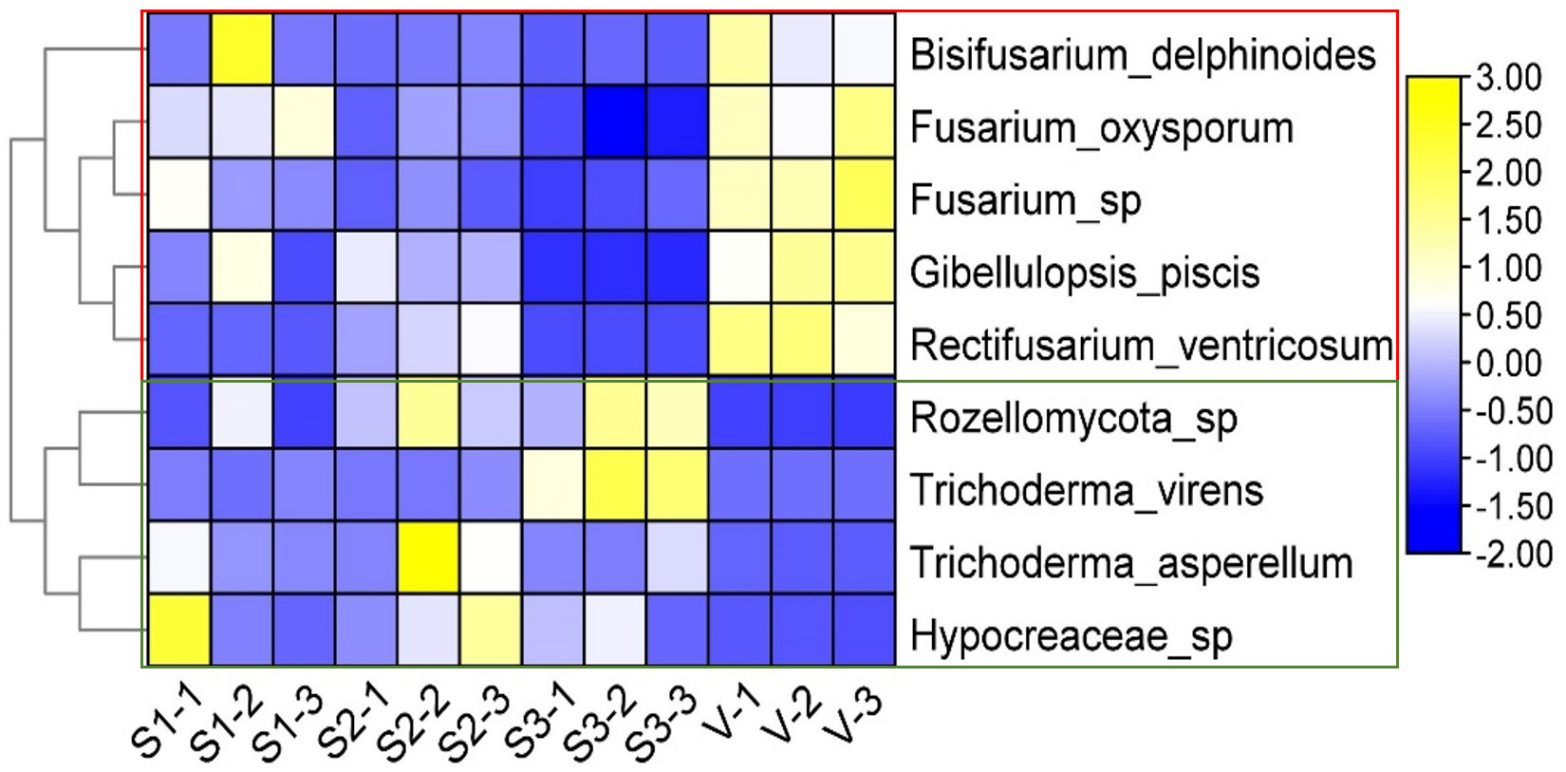
The fungal abundance and significance analysis in different banana rhizosphere soil samples. S1, S2, and S3 represent *Fusarium* wilt resistant banana plants continuously cropping for 1, 2, and 3 years, respectively. V represent for *Fusarium* wilt sensitive banana variety. Each cell represents the mean relative abundance of a fungal species (row) in a sample group (column). Color scale: blue (low abundance) to yellow (high abundance). Statistical significance was determined by *t*-test (*p* < 0.05) with FDR correction. Taxa with significantly different abundances among groups are labeled. V, susceptible; S1–S3, resistant line after 1–3 years of continuous cropping.

### 3.4 Changes in bacterial abundance in banana rhizosphere soil at different planting ages

We then performed a bacterial diversity analysis, obtaining a total of 1,278,340 effective tags in different soil samples. Contrary to the trend of fungal diversity, bacterial diversity decreased notably, but not significantly, with the increase in banana continuous cropping years. Bacterial alpha-diversity indices (Simpson, Chao1, ACE) were significantly lower in V compared to S1, S2, and S3 (*p* < 0.05, Supplementary File 1). Differential abundance analysis (*t*-test, *p* < 0.05) revealed that the susceptible variety V had significantly lower relative abundances of several potentially beneficial bacterial genera compared to the resistant line groups. Specifically, *Sphingomonas parvus* showed a marked reduction in V (0.12% ± 0.03%) compared to S3 (2.45% ± 0.31%, *p* < 0.001). Similarly, *Bryobacter*, *Reyranella*, and *Edaphobaculum* were also significantly less abundant in V. Complete pairwise comparison results are provided in Supplementary Figure 2. These findings suggest a correlation between *Fusarium* infection and reduced abundance of beneficial bacterial populations, while extended continuous cropping of the resistant line is associated with their enrichment. These findings suggest that *Fusarium* infection may suppress beneficial bacterial populations, while extended continuous cropping of the resistant line promotes their enrichment. Detailed analysis showed that banana *Fusarium* pathogens significantly reduced the abundance of probiotic bacteria such as *Sphingomonas parvus*, *Bryobacter*, *Reyranella*, and *Edaphobaculum*, especially *Sphingomonas parvus*. The results also laid the foundation for screening out the biocontrol bacteria for banana wilt resistance in the later stages.

### 3.5 Metabolome analysis of root secretions of bananas

Metabolomics was used to analyze the composition of root-secreted compounds in each group of bananas. A total of 1153 metabolic compounds were identified by widely targeted metabolomics detection, mainly including amino acids, phenolic acids, organic acids, alkaloids, flavonols, saccharides, phenolic acids, and lignans and coumarins (Figure 3A, Supplementary File 2). The quality of all detected data was evaluated in this study, including the overlap of positive and negative ion flow graphs of QC samples (Supplementary Figures 3A, B), the distribution of SV values of all sample data (Supplementary Figure 2C), and the correlation of QC samples (Supplementary Figure 3D). The results show the reliability and accuracy of the detection metabolite data in this study and can be used for further analysis. First, we performed principal component analysis (PCA) of these metabolic compounds. The results of PCA showed that each sample was significantly separated. V and S2 were significantly separated on PC1 (32.18%), and S1 and S3 were significantly separated on PC2 (24.53%) (Figure 3B). Hierarchical clustering (Figure 3C) showed that most compounds were down-regulated in V but up-regulated in S2.

**FIGURE 3:**
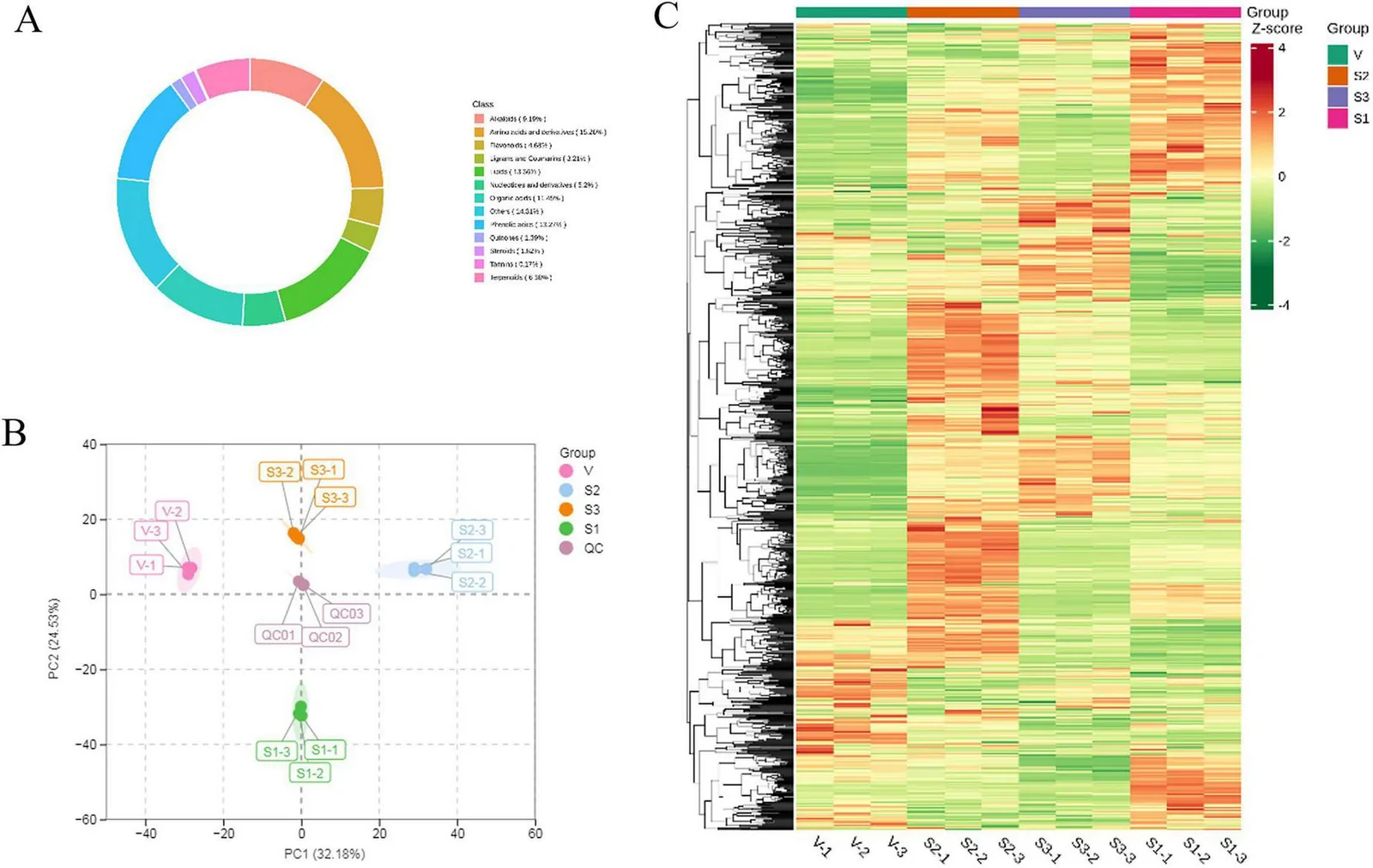
Analysis of metabolite compound classes of banana root exudates **(A)**. Principal component analysis (PCA) of metabolites detected in banana root exudates **(B)**. Hierarchical cluster analysis of all metabolites detected in banana roots **(C)**. S1, S2, and S3 represent *Fusarium* wilt resistant banana plants continuously cropping for 1, 2, and 3 years, respectively. V represent for *Fusarium* wilt sensitive banana variety. The color in the rectangular cell of the hierarchical clustering heat map identifies the level of each metabolite accumulation, with green indicating downward accumulation and red indicating upward accumulation.

### 3.6 Differentially accumulated metabolites analysis in root secretions of banana

The differentially accumulated metabolites (DAMs) of each comparison group were screened by VIP > 1, *P* < 0.05, fold change ≥ 2, and fold change ≤ 0.5 thresholds (Figure 4A). Among them, 448 DAMs (343 up-regulated and 105 down-regulated) were identified from the S1 vs. V comparison group. 465 DAMs were identified in S2 vs. V (435 up-regulated and 30 down-regulated), and 345 DAMs were identified in S3 vs. V (275 up-regulated and 70 down-regulated). The results show that, compared with V, the number of up-regulated DAMs in S1, S2, and S3 is much higher than that of down-regulated DAMs. We compare the changes in DAMs in S1, S2, and S3 by using Veen analysis. Hierarchical clustering of these shared DAMs (Figure 4C) revealed that 161 compounds were significantly increased in S1, S2, and S3 but decreased in V.

**FIGURE 4:**
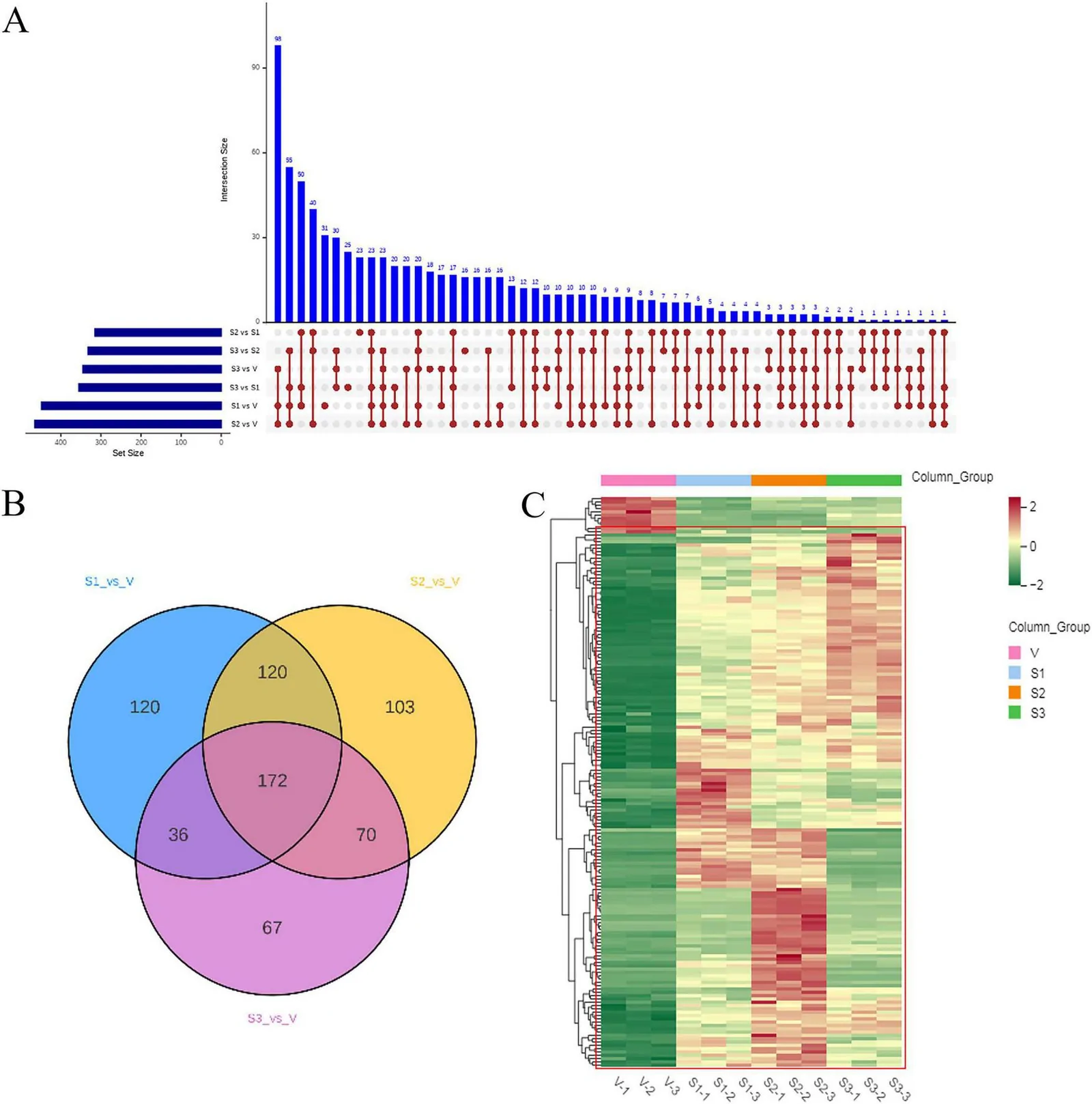
Differentially accumulated metabolites (DAMs) in banana root exudates. **(A)** Number of up-regulated and down-regulated DAMs in each comparison group (S1 vs. V, S2 vs. V, S3 vs. V). Criteria: VIP > 1, *p* < 0.05, | log2FC | ≥ 1. **(B)** Venn diagram showing the overlap of DAMs among the three comparisons. Hierarchical cluster analysis of shared DAMs identified in banana roots **(C)**. Colors: red = up-regulated; green = down-regulated. V, susceptible variety; S1, S2, S3, resistant line after 1, 2, and 3 years of continuous cropping.

Then, we used Venn analysis to compare the changes of DAMs among the three comparison groups: S1 vs. V, S2 vs. V, and S3 vs. V (Figure 4B). A total of 172 shared DAMs (including 98 specific DAMs) were selected for hierarchical cluster analysis (Figure 4C). The results showed that the concentration of 161 compounds were significantly increased in S1, S2, and S3, while significantly decreased in V, including 23 organic acids, 19 phenolic acids, 37 amino acids, 18 lipids, 5 flavonoids, and 5 lignans and coumarins, etc., (Supplementary File 3). Among them, the root exudate compounds associated with disease resistance were identified, including riboflavin, flavone, tartaric acid, coumarin, and salicylic acid. In addition, 9 compounds we screened out with the content in S1, S2, and S3 were significantly higher than that of V (more than 20 times), including 1 carbohydrate (D-mannose), 1 organic acid (3-hydroxymandelate), one coumarin (fraxetin-7, 8-di-o-glucoside), and six phenolic acids (1-dehydro-[10]-gingerdione, 3, 4-dimethoxycinnamic acid, methyl cumalate, 4-hydroxyphenylethanol, salicylic acid, and ferulic acid) (Figure 5).

**FIGURE 5:**
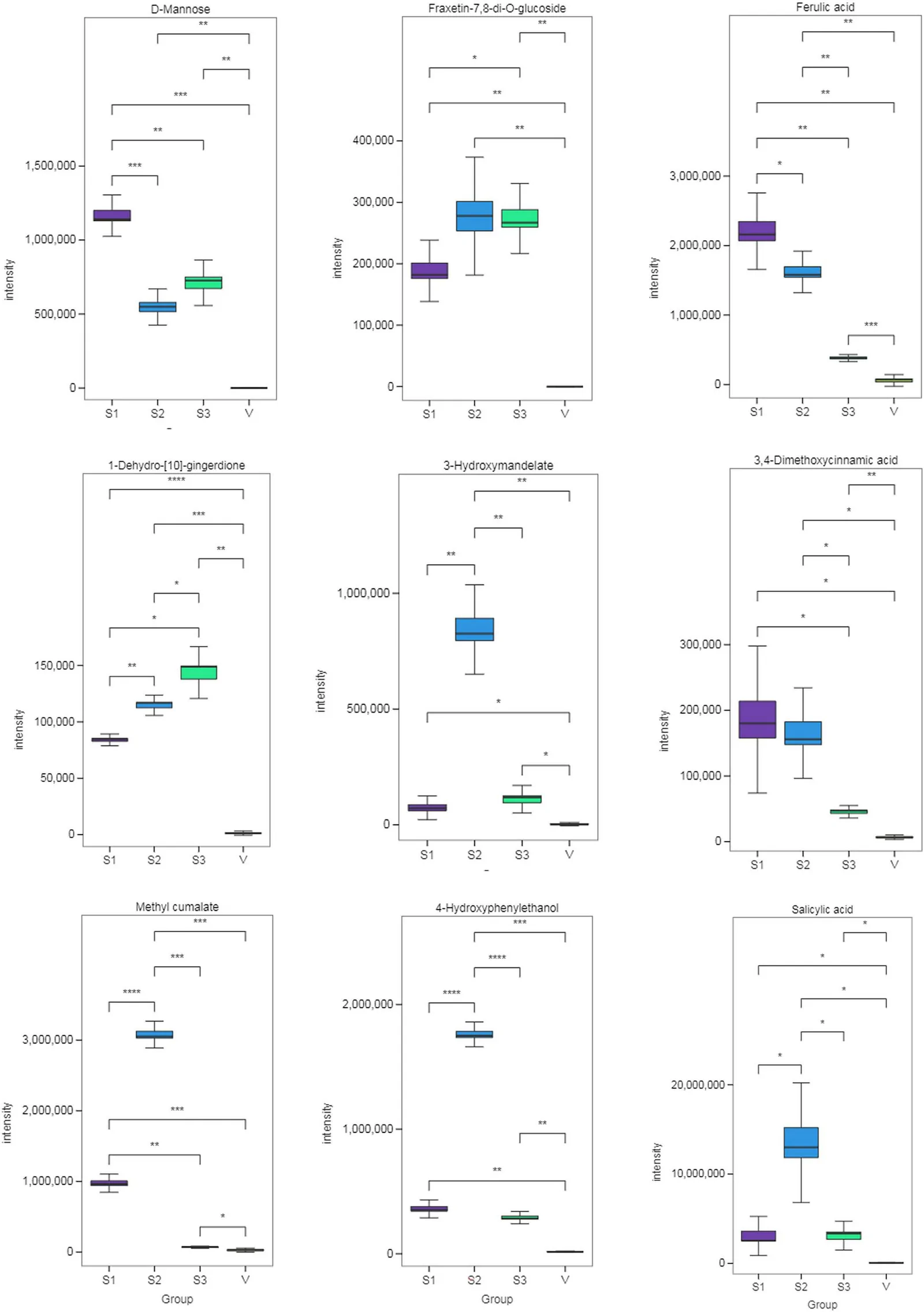
Relative contents of nine representative defense-related metabolites that were significantly higher in S1, S2, and S3 compared to V (fold change > 20). Values are means ± SD (*n* = 3). D-mannose (carbohydrate), 3-hydroxymandelate (organic acid), fraxetin-7,8-di-O-glucoside (coumarin), and six phenolic acids as indicated. Statistical significance: *p < 0.05, **p < 0.01, ***p < 0.001, ****p < 0.0001 (one-way ANOVA followed by Tukey’s test). V, susceptible; S1–S3, resistant line at increasing continuous cropping years.

### 3.7 Combined analysis of microbiome and metabolome to identify key biocontrol bacteria and metabolites in banana

Then, we conduct correlation analysis on the bacteria, fungi, and metabolites identified in each banana root soil and secretion group. A correlation network diagram is constructed by using a correlation greater than 0.6 or less than −0.6 as a threshold (Figure 6). The results showed that 9 fungi and 4 bacteria had 600 relationships with 161 metabolites. A total of 79 compounds were identified that were significantly negatively correlated with *Fusarium oxysporum* and positively correlated with Rozellomycota sp. (Supplementary File 4). Among them, it includes 8 phenolic acids (salicyl alcohol, 4-aminosalicylic acid, methyl syringate, and octyl gallate, etc.), 27 amino acids and their derivatives, 16 organic acids, etc., (Supplementary File 4). These results indicate that these metabolic compounds are positively correlated with the abundance of beneficial fungi, such as Rozellomycota. Similarly, 47 compounds were screened might as associated with *Sphingomonas parvus*, *Bryobacter* and other beneficial bacteria. This includes a large number of saccharides (Molina et al., 2022). In addition, the vitamins (pyridoxal, pyridoxine, 4-pyridoxic acid), 4 flavonoids (epigallocatechin, rhamnetin-3-O-rutinoside-5-O-rhamnoside, etc.), five phenolic acids (salicyl alcohol, (S)-2-phenyloxirane, 3,4-dimethoxycinnamic acid, ferulic acid, etc.), and eight amino acids were also associated with the enrichment of beneficial bacteria in banana rhizosphere soil with increasing continuous cropping time (Supplementary File 5).

**FIGURE 6:**
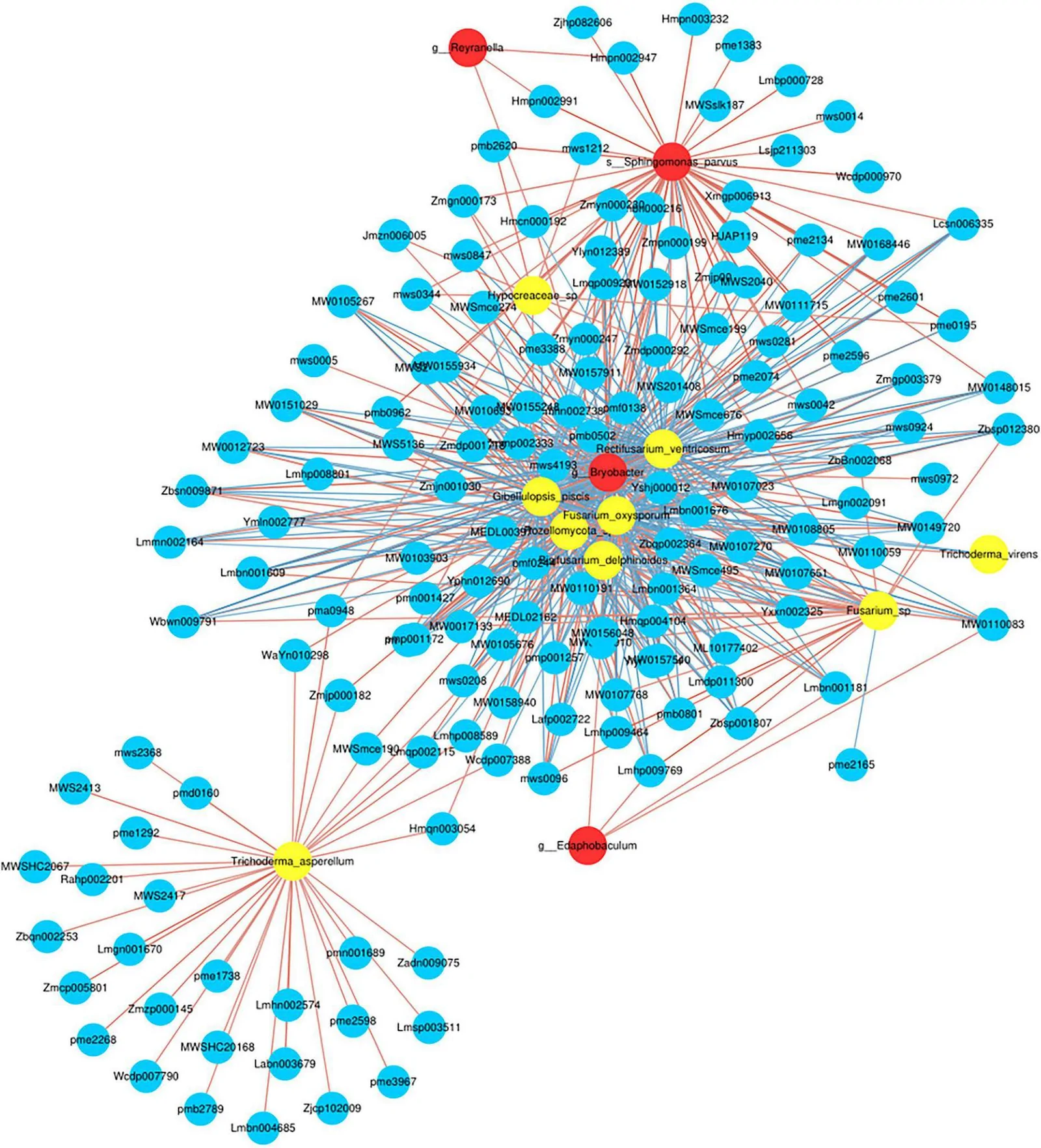
Integrated correlation network of microbiome and metabolome. Nodes: red circles = hub metabolites; yellow circles = beneficial fungi; blue circles = beneficial bacteria; green circle = pathogenic fungus (*Fusarium oxysporum*). Edges: red = positive correlation; gray = negative correlation (Spearman’s | r | > 0.6, *p* < 0.05). Node size reflects degree centrality. Only significant correlations are shown. This network illustrates those specific metabolites (e.g., salicylic acid, coumarins, amino acids) are positively associated with beneficial taxa (Rozellomycota, *Trichoderma*, *Sphingomonas*) and negatively associated with *Fusarium oxysporum*.

The primary metabolic compounds (such as amino acids, organic acids, vitamins, saccharides, etc.) secreted by banana roots play an important role in maintaining soil microbial homeostasis. However, bacteria and fungi have different requirements for these metabolites; for example, fungi prefer amino acids, organic acids, etc., while bacteria prefer saccharides, vitamins, etc. In addition, some secondary metabolic compounds, such as phenolic acids, flavonoids, coumarins and other compounds, play an important role in recruiting beneficial bacteria for inhibiting *Fusarium* pathogens.

## 4 Discussion

*Fusarium* wilt of bananas is as a pandemic vascular wilt disease. In recent years, a major spread of soil-borne fungi strains has emerged of the tropical race 4 (TR4) as the most devastating threat to bananas worldwide and well adapted to long-term survival in soil (Staver et al., 2020; Thangavelu et al., 2020). This disease infects banana cultivars’ roots, hindering water and nutrient transport, and infection of vascularized sections of the rhizome is most pronounced in susceptible genotypes (Dita et al., 2018; Ploetz, 2015). The damaged plants’ leaves yellow, typically split at the base, and eventually collapse (Pegg et al., 2019). Understanding the underlying mechanisms of plant disease resistance begins with identifying genes involved in host defense (Raman et al., 2016). The investigations of plant global gene expression patterns impacted by infections from various FOC groups that have improved our understanding of the host response to pathogen infection (Xiang et al., 2026). The results of the present study reveal an intriguing phenomenon: with increasing continuous cropping years, the incidence of *Fusarium* wilt in resistant banana varieties significantly decreased (Table 1), contrary to the conventional expectation that continuous cropping typically leads to soil degradation and increased disease pressure. This observation aligns with a parallel study by Xiang et al. (2026), who reported that resistant banana cultivars progressively assemble a stable, highly diverse corm endophytic microbiome across ratoon cycles, contributing to disease suppression (Dou et al., 2020). We hypothesize that under specific conditions, continuous cropping may be associated with the gradual establishment of a disease-suppressive rhizosphere environment, potentially linked to shifts in root exudation patterns. These changes correlate with the enrichment of beneficial microbial communities (e.g., *Trichoderma*, Rozellomycota, and *Sphingomonas*) and a decreased relative abundance of soil-borne pathogens like *Fusarium oxysporum*. However, further functional validation (e.g., isolate culturing, co-culture assays) is required to establish causal relationships. The metabolomics data support this hypothesis by showing consistent increases in beneficial taxa and defense-related metabolites (e.g., salicylic acid, coumarins, amino acids) with extended cropping duration.

*Fusarium* wilt is a key biological stress factor affecting bananas and a common disease in banana-producing locations around the world (Pegg et al., 2019). There are numerous elements that influence the presence of bananas in the field. In *Fusarium* wilt, the most important component is banana variety susceptibility, which directly impacts the host plant and its response to pathogens, while other factors, such as rhizosphere soil microbiota or plant root secretions, directly affect pathogens (Pegg et al., 2019). Therefore, in order to find the key microorganisms and metabolites that can directly affect *Fusarium* wilt pathogens, the rhizosphere soil and root secretions of banana plants in different continuous cropping years were detected by microbiome and metabolome (LC-MS/MS), respectively. Previous research has discovered that the varied stages of plant growth are critical for its resistance to sickle diseases, with mature plants being more resistant than younger plants (Husaini et al., 2018). This finding is consistent with our observation and analysis results that there is a significant positive correlation between the *Fusarium* wilt resistance of banana plants and cropping years. The *Fusarium* resistance of banana plants increased with continuous cropping time (year). These phenomena could be explained by plants actively shaping their associated microbiomes (Bressan et al., 2009; Sasse et al., 2018). The present study results also indicated that the banana wilt pathogen can affect the bacterial diversity in soil and cause the destruction of bacterial homeostasis. In the process of banana continuous cropping, the production of root exudates changes the rhizosphere soil microbial community and promotes the abundance of plant growth-promoting rhizobacteria. This can limit *Fusarium* reproduction while also increasing plant tolerance to pathogens and promoting plant growth (Turner et al., 2013).

As metabolite compounds not only support microbial life close to the plant as a source of nutrients but also act in dynamic processes of selecting and suppressing microbes for the benefit of the plant (Berendsen et al., 2012; Nazir et al., 2016), root secretion compounds are the bridge between plants, soil, and microbe assembling in plants. In this study, the types and contents of root exudates of *Fusarium* wilt-susceptible and -resistant banana plants were significantly different. In addition, we also found that the root secretion of *Fusarium* wilt-resistant plants changed significantly with the increase in continuous cropping time (year). Different root exudates have been shown to be associated with changes in the abundance and enrichment of rhizosphere colonization by microbial communities (Kudjordjie et al., 2019), despite the fact that just a modest amount of secretion chemicals is required for microbial collecting. Therefore, the results of this experiment provide correlational evidence supporting this concept. We found that there are 161 metabolic compounds, including 23 organic acids, 19 phenolic acids, 37 amino acids, 18 lipids, and 5 flavonoids, significantly accumulated in S1, S2, and S3, while significantly decreasing in V. Among them, organic acids, for example, have been shown to have a major influence on the rhizosphere microbiome, recruiting a plant growth-promoting *Pseudomonas fluorescens* strain to roots (Sood, 2003). Therefore, the riboflavin, flavone, tartaric acid, coumarin, and salicylic acid root exudates were identified as being associated with disease resistance in bananas, including D-mannose, 3-hydroxymandelate, fraxetin-7, 8-di-o-glucoside, 1-dehydro-[10]-gingerdione, 3, 4-dimethoxycinnamic acid, methyl cumalate, 4-hydroxyphenylethanol, salicylic acid, and ferulic acid. Previous research has revealed that p-coumaric acid can influence the makeup of microbial communities in the cucumber rhizosphere (Zhou and Wu, 2012). These metabolic compounds may promote the recruitment of probiotic populations. It may also be related to the inhibition of *Fusarium* wilt pathogens.

The type and quantity of root exudates influence the type and quantity of rhizosphere microorganisms, while rhizosphere microorganisms influence root exudate production (Kawasaki et al., 2016; Vora et al., 2021). In the present study, *Fusarium oxysporum*, *Bisifusarium delphinoides*, *Fusarium* sp, *Gibellulopsis piscis*, and *Rectifusarium ventricosum* were enriched in rhizosphere soil of the *Fusarium* susceptible plants compared to the resistant plants. In addition, this study also found that banana *Fusarium* pathogens were significantly associated with a lower abundance of probiotic bacteria, including *Sphingomonas parvus*, *Bryobacter*, *Reyranella*, and *Edaphobaculum*, especially *Sphingomonas parvus*. These results indicated that the root exudate profile in V was associated with a higher abundance of *Fusarium* wilt pathogens, while root exudates in S were not conducive to the reproduction of these microorganisms. This may also be the main difference between susceptible and resistant banana plants. Furthermore, Rozellomycota sp., *Trichoderma virens*, *Trichoderma asperellum*, and Hypocreaceae sp. fungi strains were identified with high abundance in S2 and S3, while they were not detected in V, which may in improving the biological prevention and control of continuous banana cultivation against pathogens. Previous research found that *Trichoderma* and Hypocreaceae had a favorable influence on plant biological control (Katoch and Pull, 2017; Yu et al., 2020), implying that these fungi could play a role in banana wilt control. The root exudates of resistant bananas may give nutrients for *Trichoderma* development and multiplicity (Qiu et al., 2018). Thus, our data suggest that the abundance of *Fusarium* pathogens in the rhizosphere soil of S banana plants was lower, and resistance was higher, in association with increased planting time. However, causal inferences await experimental validation. Colony expansion stimulated interactions among soil microflora, decreased the quantity of pathogenic *Fusarium*, and effectively regulated the occurrence of soil-borne illnesses (Vora et al., 2021).

In recent years, the outbreak of banana wilt has caused the imbalance of soil ecosystem, the increase of planting cost and the huge economic loss. Therefore, future research should continue to explore new prevention and control strategies and methods. (1) To explore efficient and broad-spectrum functional microorganisms (probiotics) to effectively inhibit the expansion and invasion of pathogens of bananas *Fusarium* wilt from biological control. (2) Deeply explore the mechanism and pathway of pathogen transmission, as well as the inhibition mechanism of biocontrol bacteria, develop more efficient and accurate pathogen detection methods, timely detect diseases and take corresponding prevention and control measures.

## Data Availability

The raw sequencing data of 16S rRNA gene and ITS amplicons have been deposited in the NCBI Sequence Read Archive (SRA) under BioProject accession number PRJNA1475535. All sequencing runs are available under corresponding SRA accession numbers (SRR39028638-SRR39028661).

## Footnotes

1
http://www.r-project.org
