# Supplementary material for: Integrated microbiome and metabolome analysis reveals mechanisms of Fusarium wilt resistance in banana rhizosphere soil under different continuous cropping durations
Source: Front Microbiol. 2026 Oct 22;17:1853743. doi: 10.3389/fmicb.2026.1853743 (PMC13736750; doi:10.3389/fmicb.2026.1853743)
Supplement: Supplementary file 1 [file Image_1.pdf]

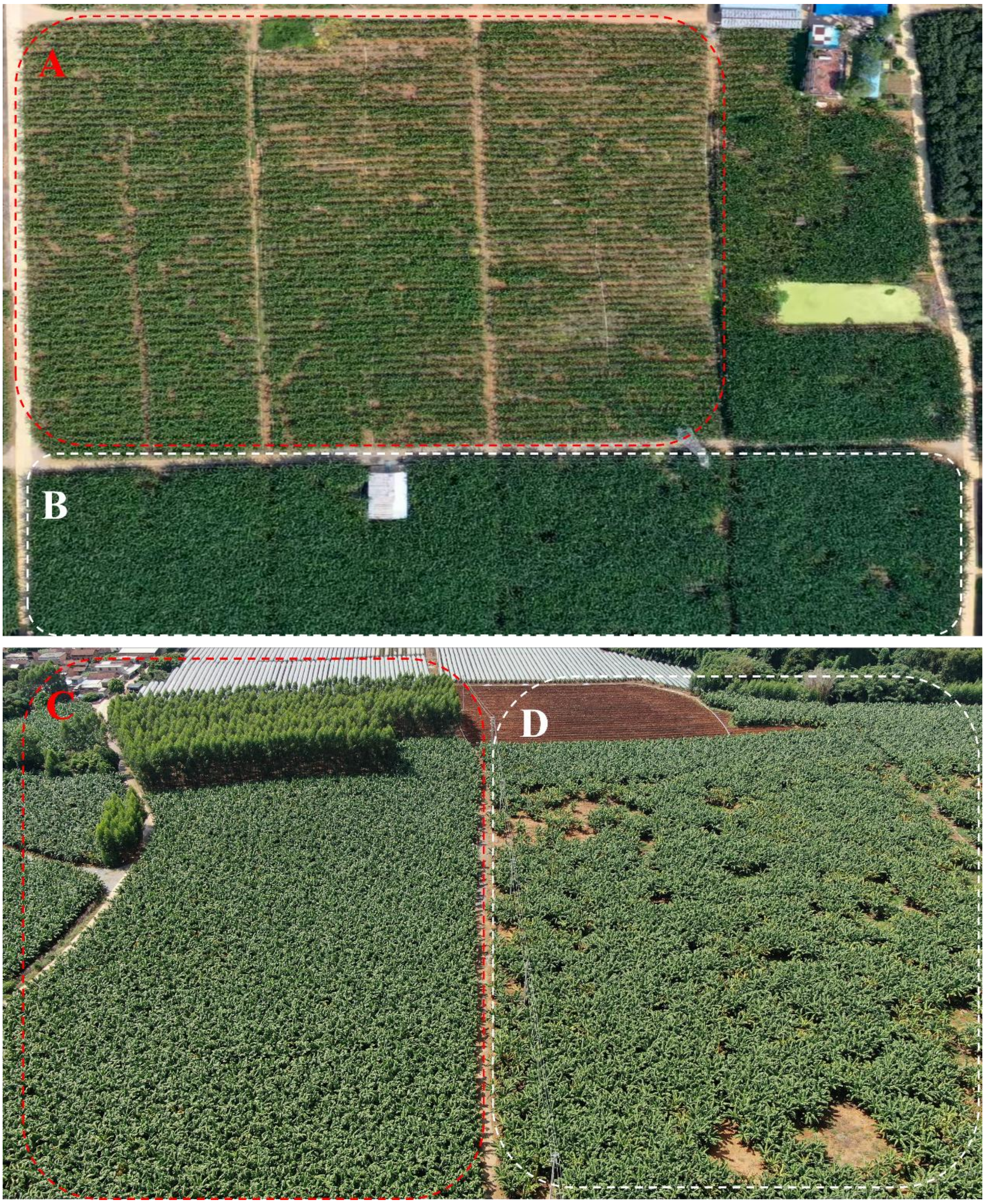

Figure S1. Phenotypic investigation of Fusarium wilt disease resistance under field cultivation. A, Newly planted first-generation 'Baodao' bananas showing extensive symptoms of Fusarium wilt. B, 'Baodao' bananas cultivated for three years exhibiting good ratooning ability and obvious disease resistance. C, Close-up view of 'Baodao' bananas cultivated for three years, displaying an enhanced resistance phenotype. D, Close-up view of 'Baodao' bananas cultivated for two years, with some plants showing symptoms of Fusarium wilt.

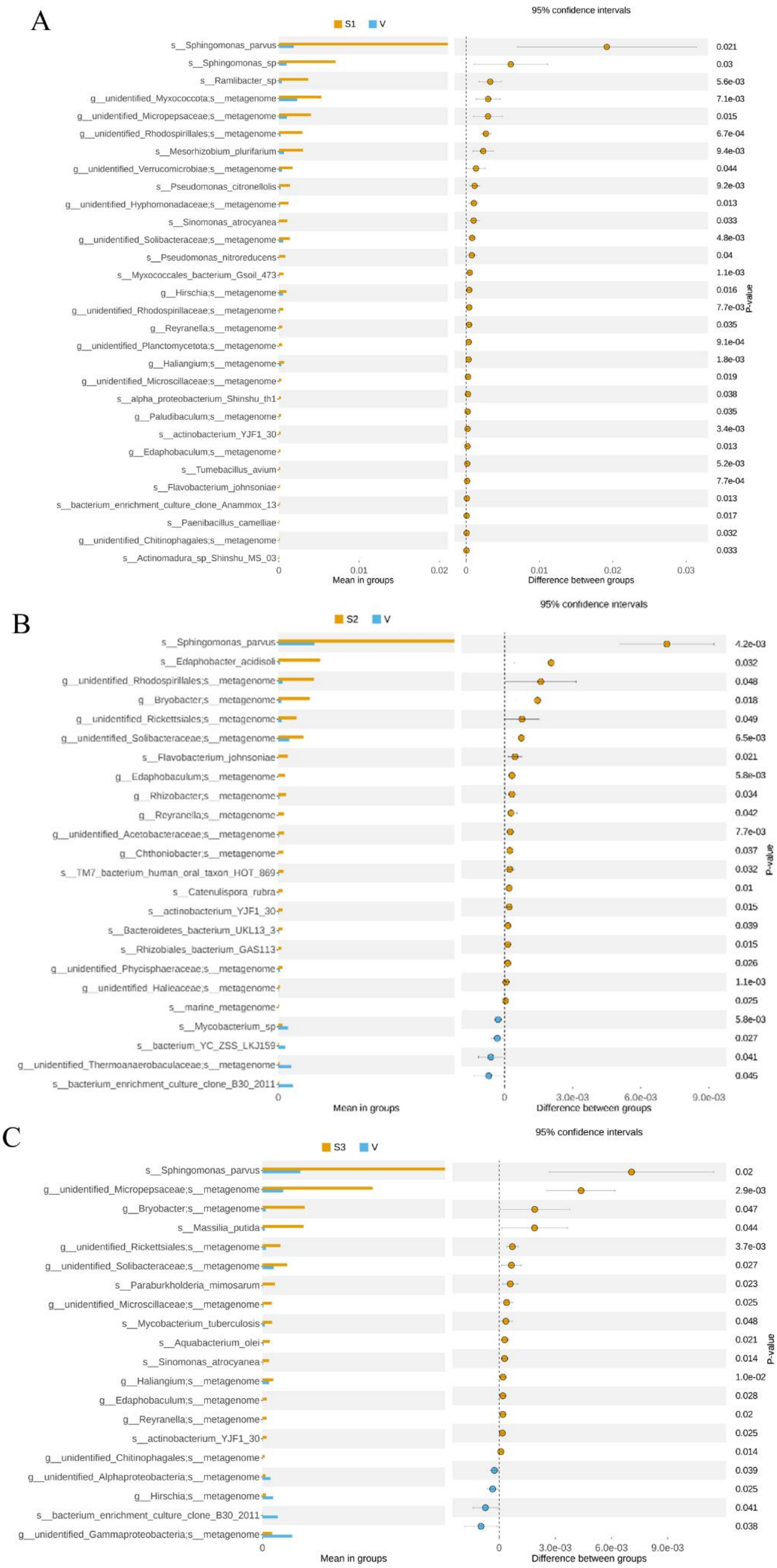

A
S1
V
95% confidence intervals
s__Sphingomonas_parvus
s__Sphingomonas_sp
s__Ramlibacter_sp
g__unidentified_Myxococcota;s__metagenome
g__unidentified_Micropepsaceae;s__metagenome
g__unidentified_Rhodospirillales;s__metagenome
s__Mesorhizobium_plurifarium
g__unidentified_Verrucomicrobiae;s__metagenome
s__Pseudomonas_citronellolis
g__unidentified_Hyphomonadaceae;s__metagenome
s__Sinomonas_atrocyanea
g__unidentified_Solibacteraceae;s__metagenome
s__Pseudomonas_nitroreducens
s__Myxococcales_bacterium_Gsoil_473
g__Hirschia;s__metagenome
g__unidentified_Rhodospirillaceae;s__metagenome
g__Reyranella;s__metagenome
g__unidentified_Planctomycetota;s__metagenome
g__Haliangium;s__metagenome
g__unidentified_Microscillaceae;s__metagenome
s__alpha_proteobacterium_Shinshu_th1
g__Paludibaculum;s__metagenome
s__actinobacterium_YJF1_30
g__Edaphobaculum;s__metagenome
s__Tumebacillus_avium
s__Flavobacterium_johnsoniae
s__bacterium_enrichment_culture_clone_Anammox_13
s__Paenibacillus_camelliae
g__unidentified_Chitinophagales;s__metagenome
s__Actinomadura_sp_Shinshu_MS_03
0.021
0.03
5.6e-03
7.1e-03
0.015
6.7e-04
9.4e-03
0.044
9.2e-03
0.013
0.033
4.8e-03
0.04
1.1e-03
0.016
7.7e-03
0.035
9.1e-04
1.8e-03
0.019
0.038
0.035
3.4e-03
0.013
5.2e-03
7.7e-04
0.013
0.017
0.032
0.033
P-value
0
0.01
0.02
Mean in groups
0
0.01
0.02
0.03
Difference between groups
B
S2
V
95% confidence intervals
s__Sphingomonas_parvus
s__Edaphobacter_acidisoli
g__unidentified_Rhodospirillales;s__metagenome
g__Bryobacter;s__metagenome
g__unidentified_Rickettsiales;s__metagenome
g__unidentified_Solibacteraceae;s__metagenome
s__Flavobacterium_johnsoniae
g__Edaphobaculum;s__metagenome
g__Rhizobacter;s__metagenome
g__Reyranella;s__metagenome
g__unidentified_Acetobacteraceae;s__metagenome
g__Chthoniobacter;s__metagenome
s__TM7_bacterium_human_oral_taxon_HOT_869
s__Catenulispora_rubra
s__actinobacterium_YJF1_30
s__Bacteroidetes_bacterium_UKL13_3
s__Rhizobiales_bacterium_GAS113
g__unidentified_Phycisphaeraceae;s__metagenome
g__unidentified_Haliceaceae;s__metagenome
s__marine_metagenome
s__Mycobacterium_sp
s__bacterium_YC_ZSS_LKJ159
g__unidentified_Thermoanaerobaculaceae;s__metagenome
s__bacterium_enrichment_culture_clone_B30_2011
4.2e-03
0.032
0.048
0.018
0.049
6.5e-03
0.021
5.8e-03
0.034
0.042
7.7e-03
0.037
0.032
0.01
0.015
0.039
0.015
0.026
1.1e-03
0.025
5.8e-03
0.027
0.041
0.045
P-value
0
Mean in groups
0
3.0e-03
6.0e-03
9.0e-03
Difference between groups
C
S3
V
95% confidence intervals
s__Sphingomonas_parvus
g__unidentified_Micropepsaceae;s__metagenome
g__Bryobacter;s__metagenome
s__Massilia_putida
g__unidentified_Rickettsiales;s__metagenome
g__unidentified_Solibacteraceae;s__metagenome
s__Paraburkholderia_mimosarum
g__unidentified_Microscillaceae;s__metagenome
s__Mycobacterium_tuberculosis
s__Aquabacterium_olei
s__Sinomonas_atrocyanea
g__Haliangium;s__metagenome
g__Edaphobaculum;s__metagenome
g__Reyranella;s__metagenome
s__actinobacterium_YJF1_30
g__unidentified_Chitinophagales;s__metagenome
g__unidentified_Alphaproteobacteria;s__metagenome
g__Hirschia;s__metagenome
s__bacterium_enrichment_culture_clone_B30_2011
g__unidentified_Gammaproteobacteria;s__metagenome
0.02
2.9e-03
0.047
0.044
3.7e-03
0.027
0.023
0.025
0.048
0.021
0.014
1.0e-02
0.028
0.02
0.025
0.014
0.039
0.025
0.041
0.038
P-value
0
Mean in groups
0
3.0e-03
6.0e-03
9.0e-03
Difference between groups

Figure S2. ASV based T-test analysis of the bacterial diversity in different banana rhizosphere soil samples. The figure on the left shows the abundance of different species between each group. Each bar in the figure represents the mean value of species with significant abundance differences between groups in each group. The leftmost endpoint of each circle in the graph represents the lower limit of the 95% confidence interval for the mean difference, and the rightmost endpoint of the circle represents the upper limit of the 95% confidence interval for the mean difference. The center of a circle represents the difference between the means. At the far-right end of the results presented are the p values of the inter-group significance test for the different species.

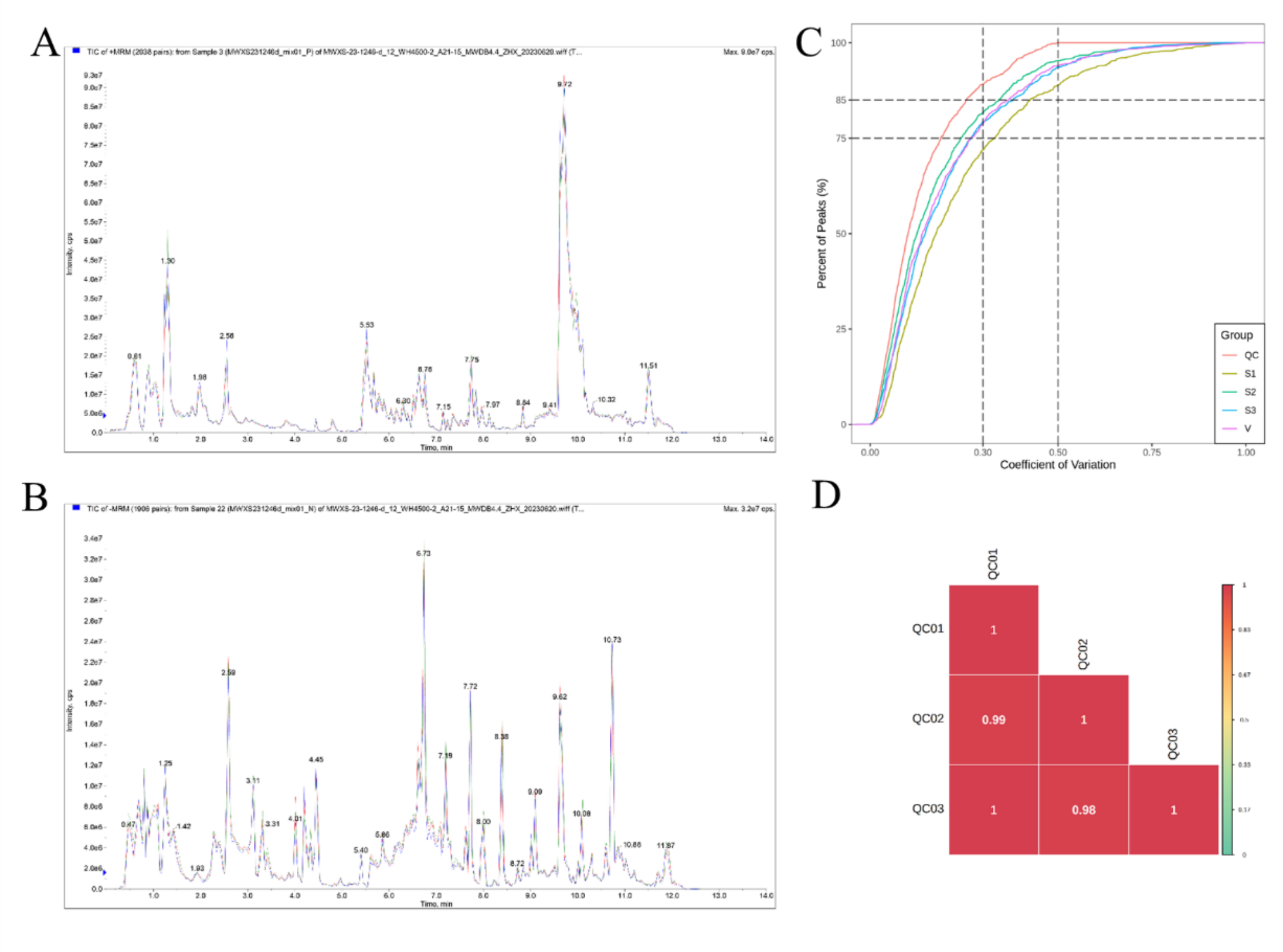

Figure S3. The overlap of positive (A) and negative (B) ion flow graphs of QC samples. The distribution of SV values of all sample data (C). The correlation of QC samples (D).
