# Supplementary material for: Integrated microbiome and metabolome analysis reveals mechanisms of Fusarium wilt resistance in banana rhizosphere soil under different continuous cropping durations
Source: Front Microbiol. 2026 Oct 22;17:1853743. doi: 10.3389/fmicb.2026.1853743 (PMC13736750; doi:10.3389/fmicb.2026.1853743)
Supplement: Supplementary file 2 [file Table_1.docx]

Table S1. The details of fungal abundance in V, S1, S2, and S3 banana rhizosphere soil samples.

| Taxonomy | S1-1 | S1-2 | S1-3 | S2-1 | S2-2 | S2-3 | S3-1 | S3-2 | S3-3 | V-1 | V-2 | V-3 |
| --- | --- | --- | --- | --- | --- | --- | --- | --- | --- | --- | --- | --- |
| *Fusarium oxysporum* | 0.0638 | 0.0661 | 0.0780 | 0.0401 | 0.0525 | 0.0501 | 0.0363 | 0.0215 | 0.0268 | 0.0832 | 0.0703 | 0.0954 |
| *Rozellomycota sp.* | 0.0043 | 0.0263 | 0.0015 | 0.0194 | 0.0429 | 0.0205 | 0.0172 | 0.0432 | 0.0375 | 0.0015 | 0.0011 | 0.0006 |
| *Trichoderma asperellum* | 0.0134 | 0.0053 | 0.0042 | 0.0038 | 0.0354 | 0.0142 | 0.0039 | 0.0033 | 0.0110 | 0.0012 | 0.0005 | 0.0005 |
| *Trichoderma virens* | 0.0002 | 0.0000 | 0.0004 | 0.0001 | 0.0001 | 0.0005 | 0.0029 | 0.0054 | 0.0048 | 0.0000 | 0.0000 | 0.0000 |
| *Fusarium sp.* | 0.0276 | 0.0134 | 0.0114 | 0.0057 | 0.0121 | 0.0051 | 0.0013 | 0.0034 | 0.0070 | 0.0349 | 0.0364 | 0.0487 |
| *Gibellulopsis piscis* | 0.0087 | 0.0223 | 0.0037 | 0.0180 | 0.0128 | 0.0129 | 0.0011 | 0.0009 | 0.0004 | 0.0203 | 0.0292 | 0.0300 |
| *Rectifusarium ventricosum* | 0.0002 | 0.0002 | 0.0001 | 0.0009 | 0.0013 | 0.0017 | 0.0000 | 0.0000 | 0.0000 | 0.0029 | 0.0031 | 0.0021 |
| *Bisifusarium delphinoides* | 0.0015 | 0.0187 | 0.0013 | 0.0009 | 0.0015 | 0.0020 | 0.0000 | 0.0005 | 0.0000 | 0.0125 | 0.0071 | 0.0077 |
| *Hypocreaceae sp.* | 0.0326 | 0.0044 | 0.0021 | 0.0055 | 0.0131 | 0.0237 | 0.0099 | 0.0140 | 0.0021 | 0.0010 | 0.0007 | 0.0001 |
